# Supplementary material for: Quantitative characterization of all single amino acid variants of a viral capsid-based drug delivery vehicle
Source: Nat Commun. 2018 Apr 11;9:1385. doi: 10.1038/s41467-018-03783-y (PMC5895741; doi:10.1038/s41467-018-03783-y)
Supplement: Supplementary file 1 — Supplementary Information(PDF 5872 kb) [file 41467_2018_3783_MOESM1_ESM.pdf]

Supplementary Information

Quantitative characterization of all single amino acid variants of a viral capsid-based drug delivery vehicle

Hartman, et al.

Supplementary Methods

Apparent Fitness Landscape (AFL) Definitions

m: one of the 21 mutations encoded by the NNK codon, including the 20 canonical amino acids and a stop codon.
$m \in$ {A, S, T, V, C, E, D, K, R, Q, N, M, I, L, H, F, Y, W, G, P, * }

$A_{p,m}$: an abundance score, indexed by position, p, and mutation, m.

CA_I : position indices corresponding to each entry vector, where CA_1 = 1-26, CA_2 = 27-52, CA_3 = 53-78, CA_4 = 79-104, CA_5 = 105-129.

$PA_{I,p,m}$: a percent abundance score, indexed by position, p, and mutation, m, within positional indices, i.

$RPA_{R,p,m}$: a relative percent abundance score, indexed by position, p, and mutation, m, corresponding to replicate, R.

$f_{p,m}$: a fitness score, indexed by position, p, and mutation, m.

Apparent Fitness Landscape (AFL) Calculations

Aligned, trimmed sequences were analyzed with code written in-house. Following data processing described above, a textfile was produced for each experiment that contains one sequencing read per line. This file was read into python, and lines that did not begin with ATG and end with TAA were discarded.

Each line was compared to the wild-type MS2 CP, and the total number of codons containing mutations was counted. Wild-type reads, or lines without any mutations, were discarded. If more than one codon in a given line contained a mutation, then any codons with single base pair mutations (as opposed to 2- or 3- base pair changes to a single codon) were assumed to be sequencing errors and discarded. Lines with one mutated codon were kept.

Every non-wild-type codon in every read was counted into a codon abundance array. Codons that did not correspond to NNK codons, meaning, the codon ended in either C or A, were discarded. The remaining codons were translated and combined by amino acid identity to generate A.

We divided A into five submatrices corresponding to the length of each primer set (see EMPIRIC cloning). These submatrices are represented by matrices A_I. The grand sum, or the sum of all counts at every amino acid along every position, was calculated:

$$CA_I = \sum A_{I,p,m}$$

We next divided A_I by its grand sum CA_I, generating a matrix of percent abundances, PA_I:

$$PA_I = \left(\frac{A_I}{CA_I}\right)$$

The submatrices were remerged into a parent percent abundance array, $PA \in \mathbb{R}^{129\times21}$. These calculations were repeated for each biological replicate of VLP and plasmid libraries, generating six PA

matrices ($PA_{R,L}$) where R indicates biological replicate, and L indicates either the VLP (V) or Unselected (U) library. We calculated relative percent abundances, $RPA_{R,p,m}$ by dividing $PA_{R,V}$ by $PA_{R,U}$ for each replicate:

$$RPA_R = \left(\frac{PA_{R,V}}{PA_{R,U}}\right)$$

We calculated the mean and standard deviation across three RPA replicates. All nan values, which indicate variants that were not identified in the plasmid library, were ignored. Scores of zero, which indicate variants that were sequenced in the Unselected library but absent in the VLP library, were replaced with an arbitrary score of 0.0001:

$$RPA_{p,m} = \left(max(RPA_{p,m}, 0.0001)\right)$$

We calculated the log10 of the RPA array to calculate the final $f_{p,m}$ array.

$$f_{p,m} = log_{10}(RPA_{p,m})$$

$f_{p,m}$ is plotted in Figure 2 and is available as a supplemental csv file. Error was propagated to generate standard deviations, which are plotted in Figure S2:

$$\sigma(f_{p,m}) = \left(0.434\frac{\sigma(RPA_{p,m})}{\mu(RPA_{p,m})}\right)$$

We calculated the average AFS value for each amino acid by finding the mean $f_{p,m}$ value for every mutation, m. These values are plotted in Figure 7a. Error indicates SEM values.

Mutability Index Definitions

Shannon Entropy can be used to calculate diversity at a given residue. Here, differential Shannon Entropy is determined to generate a Mutability Index, or MI.

$P_{p,m}$: a probability score, indexed by position, p, and mutation, m.

SE_p : a Shannon Entropy score, indexed by position.

MI_p : an score of mutability, indexed by position.

Mutability Index Calculations

Shannon entropy is defined as:

$$ShannonEntropy = -\sum P\log(P)$$

where P refers to a given probability. We first calculate the probability of a given codon occurring within a single residue:

$$P_{p,m} = \frac{A_{p,m}}{\sum(A_p)}$$

Any zero values in the resulting average array were replaced with .00001.

$$P_{p,m} = max(P_{p,m}, .00001)$$

We calculated the Shannon entropy at every residue, generating $SE \in \mathbb{R}^{129\times1}$.

$$SE_p = -\sum P_{p,m} \cdot log_{10}(P_{p,m})$$

Shannon Entropy values were averaged across three biological replicates for each library. The difference between the Unselected plasmid library (U) and the VLP library (V) generated the Mutability Index, $MI \in \mathbb{R}^{129\times1}$, which is used as a proxy for mutability and is available as a supplemental csv file.

$$MI = SE_V - SE_U$$

Average Mutability Clustering Definitions

$\mu_{n\to m}$: the average value of all fitness scores corresponding to substituting a native residue, n , with a mutant residue, m , averaged over all occurrences of such a mutation throughout the protein backbone.

$n \in$ {A, S, T, V, C, E, D, K, R, Q, N, M, I, L, F, Y, W, G, P }

It is worth noting that the set of native residues, n, does not contain histidine as there is no native occurrence of histidine in MS2's backbone

$m \in$ {A, S, T, V, C, E, D, K, R, Q, N, M, I, L, H, F, Y, W, G, P, * }

In consistency with our other analyses, the symbol " * " here represents a mutation to a stop-codon.

Average Mutability Clustering Analysis

We begin by constructing an array, Υ, containing average substitution scores, $\mu_{n\to m}$, where rows correspond to native residues, and columns correspond to the substituted residue:

$$
\Upsilon = \begin{bmatrix}
\mu_{A\to A} & \mu_{A\to S} & \mu_{A\to T} & \mu_{A\to V} & \cdots & \mu_{A\to W} & \mu_{A\to G} & \mu_{A\to P} & \mu_{A\to *} \\
\mu_{S\to A} & \mu_{S\to S} & \mu_{S\to T} & \cdots & & \cdots & \mu_{S\to G} & \mu_{S\to P} & \mu_{S\to *} \\
\mu_{T\to A} & \mu_{T\to S} & & & & & & \mu_{T\to P} & \mu_{T\to *} \\
\mu_{V\to A} & \vdots & & \ddots & & & & \vdots & \mu_{V\to *} \\
\vdots & & & & \ddots & & & & \vdots \\
\mu_{Y\to A} & \vdots & & & & \ddots & & \vdots & \mu_{Y\to *} \\
\mu_{W\to A} & \mu_{W\to S} & & & & & & \mu_{W\to P} & \mu_{W\to *} \\
\mu_{G\to A} & \mu_{G\to S} & \mu_{G\to T} & \cdots & & \cdots & \mu_{G\to G} & \mu_{G\to P} & \mu_{G\to *} \\
\mu_{P\to A} & \mu_{P\to S} & \mu_{P\to T} & \mu_{P\to V} & \cdots & \mu_{P\to W} & \mu_{P\to G} & \mu_{P\to P} & \mu_{P\to *}
\end{bmatrix}_{19\times 21}
$$

We then input this array into MATLAB's "clustergram" function, to create a graphical object (visualized in Figure 7b) via a hierarchal clustering algorithm:

This hierarchal clustering algorithm sequentially reorders all columns and rows (respectively) from the input array in a manner which minimizes the difference between values in adjacent array entries – where the "difference" between the values of array entries is defined by a two-dimensional euclidean distance calculation.

Physical Property Preference Definitions

Physical properties for all amino acids were obtained from previous literature[5-13]. These values were tabulated and normalized to between 0 and 1 to allow comparison of relative preference, and apparent fitness scores were normalized from -1 to 1. The tolerance of a given residue for each physical property was obtained by the summation of the fitness for every amino acid multiplied by its normalized physical value. This results in an overall negative score for residues where the given property is detrimental (such as highly polar amino acids in hydrophobic regions) and a positive score if the property is well tolerated (such as residue flexibility in loop regions).

a: one of the 20 canonical amino acids,
$a \in$ {A, S, T, V, C, E, D, K, R, Q, N, M, I, L, H, F, Y, W, G, P }

ε: one of the 10 physical property indices used in our analysis
(volume, molecular weight, length, sterics, polarity, polar area, fraction water, hydrophobicity, non-polar area, flexibility)

$f_{p,a}$: a fitness scores, indexed by position, p, and amino acid, a.

R_p : a vector containing the fitness scores of each amino acid for a given position, p

$$R_p = \begin{bmatrix} f_{A,p} & f_{S,p} & f_{T,p} & \cdots & f_{G,p} & f_{P,p} \end{bmatrix}_{1\times 20}$$

ξ_ε : a vector containing the physical property indices, $\varphi_{a,\varepsilon}$ corresponding to a given property, ε, and amino acid, a.

$$\xi_\varepsilon = \begin{bmatrix} \varphi_{A,\varepsilon} & \varphi_{S,\varepsilon} & \varphi_{T,\varepsilon} & \cdots & \varphi_{G,\varepsilon} & \varphi_{P,\varepsilon} \end{bmatrix}_{1\times 20}$$

$\mu(R_p)$: the mean value of the fitness scores for a given position

$\sigma(R_p)$: the standard deviation of the fitness scores for a given position

$\mu(\xi_\varepsilon)$: the mean value of the amino acid indices for a given physical property

$\sigma(\xi_\varepsilon)$: the standard deviation of the amino acid indices for a given physical property

Physical Property Preference Standardization

We proceed to produce standardized fitness scores, $\tilde{f}_{p,a}$, by taking the difference from a given position's mean, and dividing by a position's standard deviation:

$$\tilde{f}_{p,a} = \left(\frac{f_{p,a} - \mu(R_p)}{\sigma(R_p)} \right)$$

Combining these standardized fitness scores into an array, $F \in \mathbb{R}^{129\times 20}$

$$F = \begin{bmatrix} \left(\frac{f_{1,A}-\mu_1}{\sigma_1}\right) & \left(\frac{f_{1,S}-\mu_1}{\sigma_1}\right) & \cdots & \left(\frac{f_{1,P}-\mu_1}{\sigma_1}\right) \\ \left(\frac{f_{2,A}-\mu_2}{\sigma_2}\right) & \left(\frac{f_{2,S}-\mu_2}{\sigma_2}\right) & & \left(\frac{f_{2,P}-\mu_2}{\sigma_2}\right) \\ \vdots & & \ddots & \vdots \\ \left(\frac{f_{128,A}-\mu_{128}}{\sigma_{128}}\right) & \left(\frac{f_{128,S}-\mu_{128}}{\sigma_{128}}\right) & & \left(\frac{f_{128,P}-\mu_{128}}{\sigma_{128}}\right) \\ \left(\frac{f_{129,A}-\mu_{129}}{\sigma_{129}}\right) & \left(\frac{f_{129,S}-\mu_{129}}{\sigma_{129}}\right) & \cdots & \left(\frac{f_{129,P}-\mu_{129}}{\sigma_{129}}\right) \end{bmatrix}_{129\times 20}$$

$$= \begin{bmatrix} \tilde{f}_{A,1} & \tilde{f}_{S,1} & \cdots & \tilde{f}_{G,1} & \tilde{f}_{P,1} \\ \tilde{f}_{A,2} & & & & \tilde{f}_{P,2} \\ \vdots & & \ddots & & \vdots \\ \tilde{f}_{A,128} & & & & \tilde{f}_{P,128} \\ \tilde{f}_{A,129} & \tilde{f}_{S,129} & \cdots & \tilde{f}_{G,129} & \tilde{f}_{P,129} \end{bmatrix}_{129\times 20}$$

Similarly, we produced standardized property indices $[\varphi_{a,\varepsilon}]_{scaled,0}$, by taking the difference from a given property's mean index value, and dividing by the associated standard deviation:

$$[\varphi_{a,\varepsilon}]_{scaled,0} = \left(\frac{\varphi_{a,\varepsilon} - \mu(\xi_\varepsilon)}{\sigma(\xi_\varepsilon)} \right)$$

Next, we subtracted the minimum value of $[\varphi_{a,\varepsilon}]_{scaled,0}$ for a given property, setting the minimum value to zero:

$$[\varphi_{a,\varepsilon}]_{scaled,1} = [\varphi_{a,\varepsilon}]_{scaled,0} - min\left\{[\varphi_{A,\varepsilon}]_{scaled,0}\,,\ [\varphi_{S,\varepsilon}]_{scaled,0}\,,\ \cdots\,,\ [\varphi_{P,\varepsilon}]_{scaled,0}\right\}$$

Finally, we divide each value of $[\varphi_{a,\varepsilon}]_{scaled,1}$ for a given property by the maximum value of its associated set, thus setting the max value to 1, and producing a set of standardized indices, $\tilde{\varphi}_{a,\varepsilon}$, fit between 0 and 1:

$$\tilde{\varphi}_{a,\varepsilon} = \left(\frac{[\varphi_{a,\varepsilon}]_{scaled,1}}{max\left\{[\varphi_{A,\varepsilon}]_{scaled,0}\,,\ [\varphi_{S,\varepsilon}]_{scaled,0}\,,\ \cdots\,,\ [\varphi_{P,\varepsilon}]_{scaled,0}\right\}} \right)$$

Combining these standardized indices into an array, $\Phi \in \mathbb{R}^{10\times 20}$

$$\Phi = \begin{bmatrix} \tilde{\varphi}_{A,volume} & \tilde{\varphi}_{S,volume} & \cdots & \tilde{\varphi}_{G,volume} & \tilde{\varphi}_{P,volume} \\ \tilde{\varphi}_{A,weight} & & & & \tilde{\varphi}_{P,weight} \\ \vdots & & \ddots & & \vdots \\ \tilde{\varphi}_{A,\ n.p.-area} & & & & \tilde{\varphi}_{P,\ n.p.-area} \\ \tilde{\varphi}_{A,flexibility} & \tilde{\varphi}_{S,flexibility} & \cdots & \tilde{\varphi}_{G,flexibility} & \tilde{\varphi}_{P,flexibility} \end{bmatrix}_{10\times 20}$$

Physical Property Preference Calculations

We can produce an array, $\Psi \in \mathbb{R}^{129\times 10}$, with entries representing each position's preference for a given physical property by the following operation:

$$\Psi = F \cdot \Phi^T$$

With individual entries, $\psi_{p,\epsilon}$, corresponding to a summation of the following product over all 20 canonical amino acids, where ϵ corresponds to a given physical property, and p corresponds to a position in the protein backbone:

$$\psi_{p,\epsilon} = \sum_{i=1}^{20} \tilde{f}_{p,a_i} \cdot \tilde{\varphi}_{a_i,\epsilon}$$

Such that:

$$\Psi = \begin{bmatrix} \left(\sum_{i=1}^{20} \tilde{f}_{1,a_i} \cdot \tilde{\varphi}_{a_i,vol.}\right) & \cdots & \left(\sum_{i=1}^{20} \tilde{f}_{1,a_i} \cdot \tilde{\varphi}_{a_i,flex.}\right) \\ \vdots & \ddots & \vdots \\ \left(\sum_{i=1}^{20} \tilde{f}_{129,a_i} \cdot \tilde{\varphi}_{a_i,vol.}\right) & \cdots & \left(\sum_{i=1}^{20} \tilde{f}_{129,a_i} \cdot \tilde{\varphi}_{a_i,flex.}\right) \end{bmatrix}_{129\times 10}$$

$$= \begin{bmatrix} \left(\tilde{f}_{A,1} \cdot \tilde{\varphi}_{A,vol.} + \cdots + \tilde{f}_{P,1} \cdot \tilde{\varphi}_{P,vol.}\right) & \cdots & \left(\tilde{f}_{A,1} \cdot \tilde{\varphi}_{A,flex.} + \cdots + \tilde{f}_{P,1} \cdot \tilde{\varphi}_{P,flex.}\right) \\ \vdots & \ddots & \vdots \\ \left(\tilde{f}_{A,129} \cdot \tilde{\varphi}_{A,vol.} + \cdots + \tilde{f}_{P,129} \cdot \tilde{\varphi}_{P,vol.}\right) & \cdots & \left(\tilde{f}_{A,129} \cdot \tilde{\varphi}_{A,flex.} + \cdots + \tilde{f}_{P,129} \cdot \tilde{\varphi}_{P,flex.}\right) \end{bmatrix}_{129\times 10}$$

$$= \begin{bmatrix} \psi_{1,volume} & \psi_{1,weight} & \cdots & \psi_{1,n.p.\ area} & \psi_{1,flexibility} \\ \psi_{2,volume} & & & & \psi_{p,flexibility} \\ \vdots & & \ddots & & \vdots \\ \psi_{128,volume} & & & & \psi_{128,flexibility} \\ \psi_{129,volume} & \psi_{129,weight} & \cdots & \psi_{129,n.p\ area} & \psi_{129,flexibility} \end{bmatrix}_{129\times 10}$$

Thus, we have obtained an array containing information about position-wise preferences for various physical properties, wherein rows index to the positions in the protein backbone, and columns index the various physical properties in question.

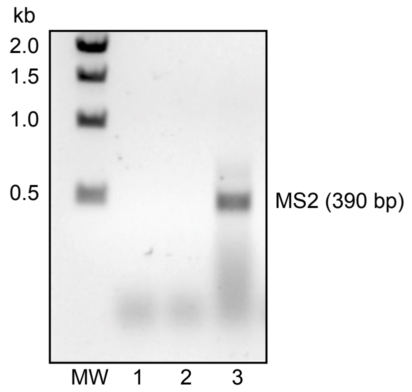

Supplementary Figure 1. CP[WT], CP[T19Stop], and CP[Non-assembling] VLPs were tested for RNA after the selection process using PCR. Lane 1: CP[T19Stop]; Lane 2: CP[Non-assembling]; Lane 3: CP[WT].

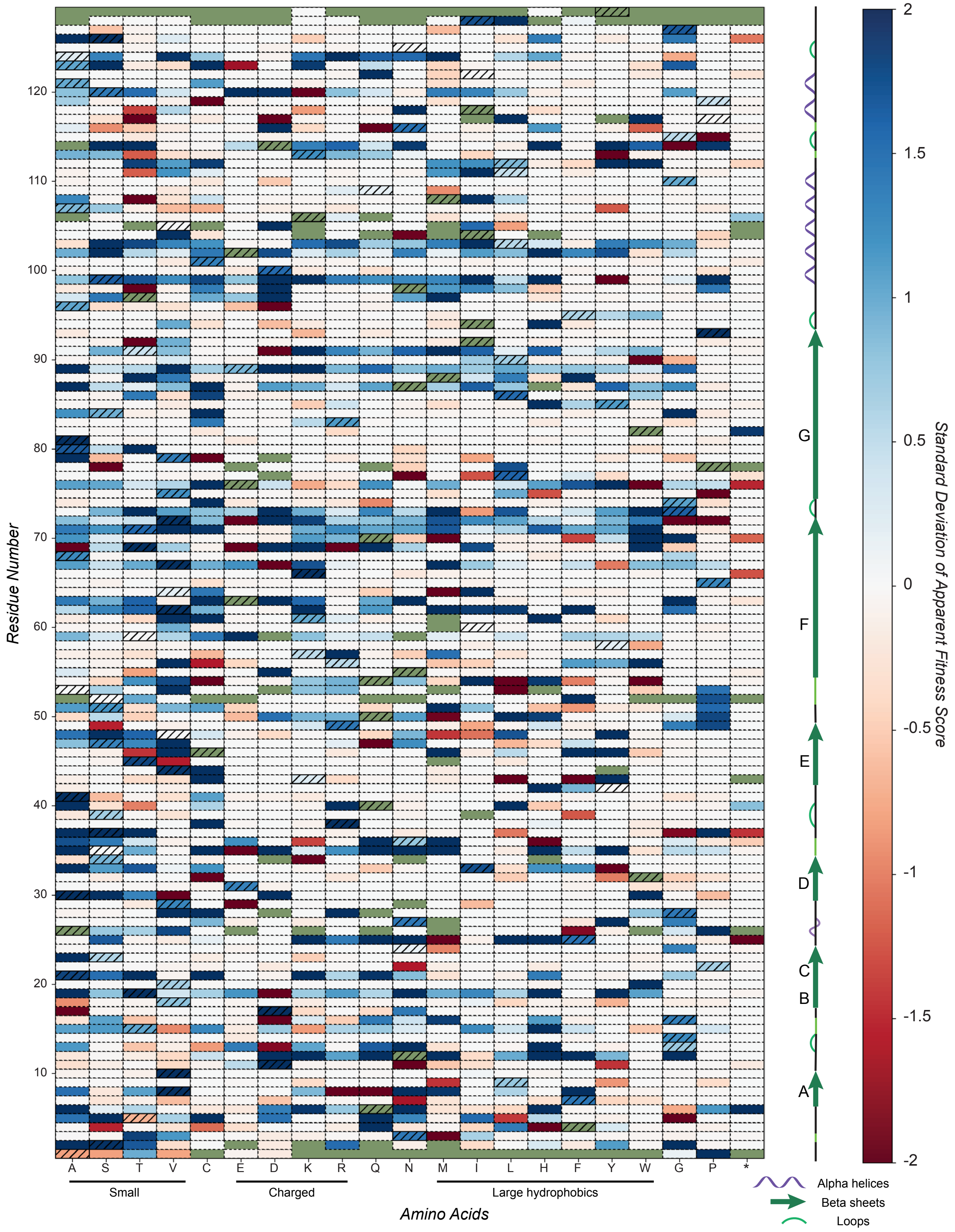

Supplementary Figure 2. Standard deviations of Apparent Fitness Scores (n=3). Logarithmic values are reported, where blue values are large standard deviations and red values are small standard deviations. Wild-type residues are indicated with hatches, and missing values are green.

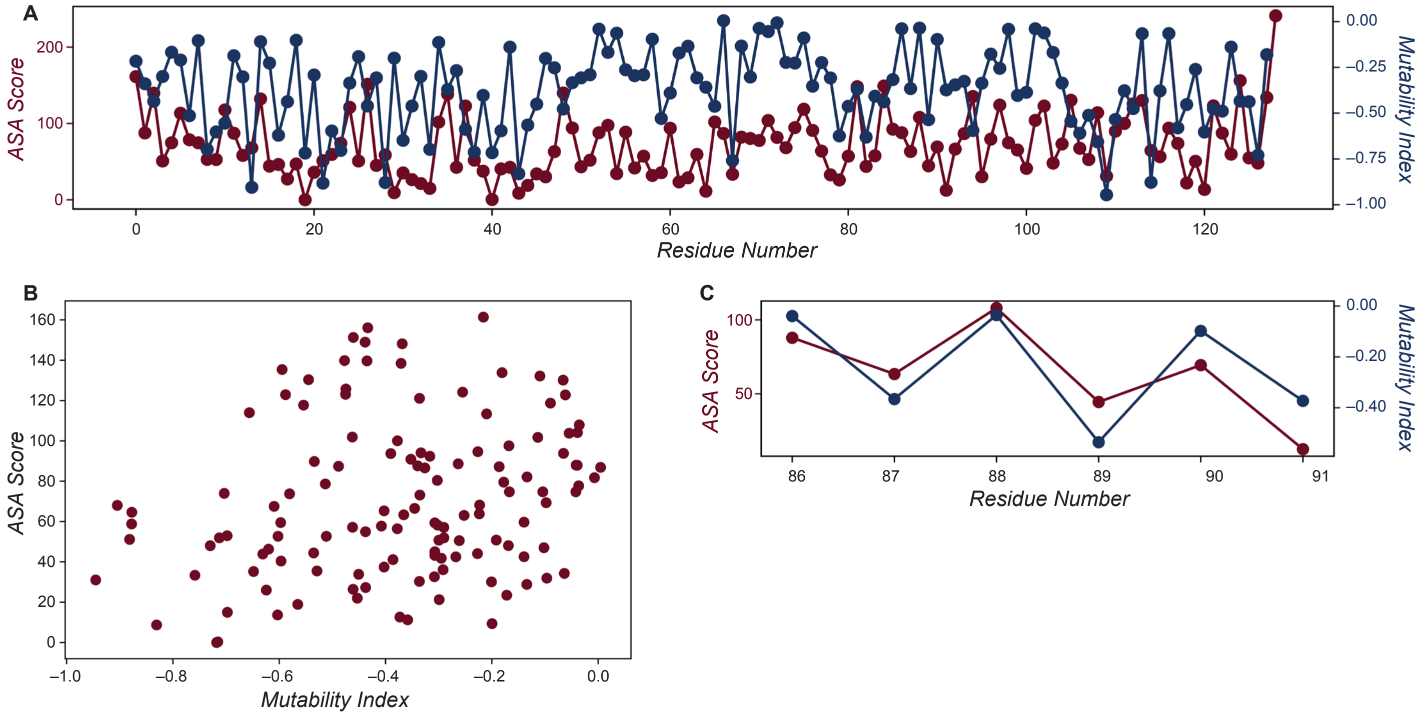

Supplementary Figure 3. Accessible surface area (ASA) compared to Mutability Index (MI). The ASA scores and MI values are plotted A) by residue number, and B) as a scatterplot. C) Beta sheet G ASA values are compared to the MI values for each residue.

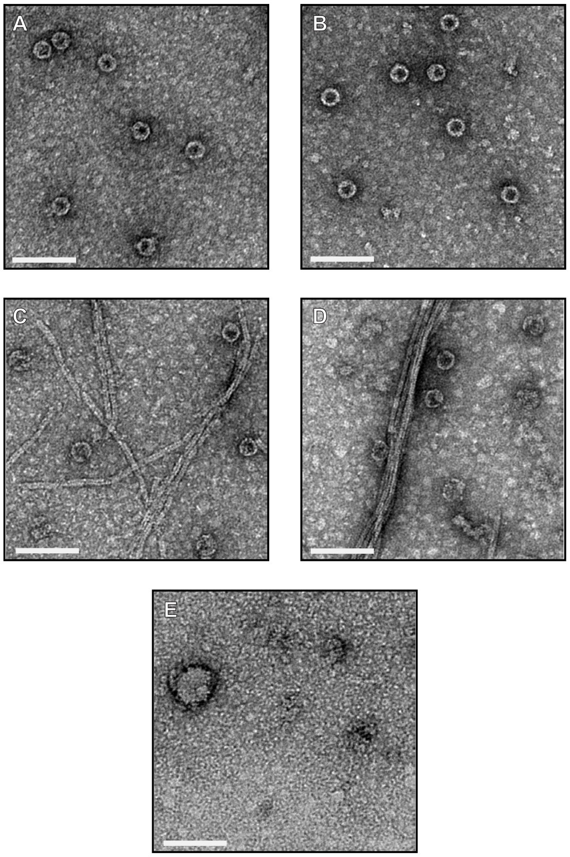

Supplementary Figure 4. TEM images of MS2 VLPs. A) CP[WT], B) CP[T71H], C) CP[T91C], D) CP[Q50C], and E) CP[F4V] are imaged using a $UO_2(Ac)_2$ negative stain. Scale bars indicate 100 nm.

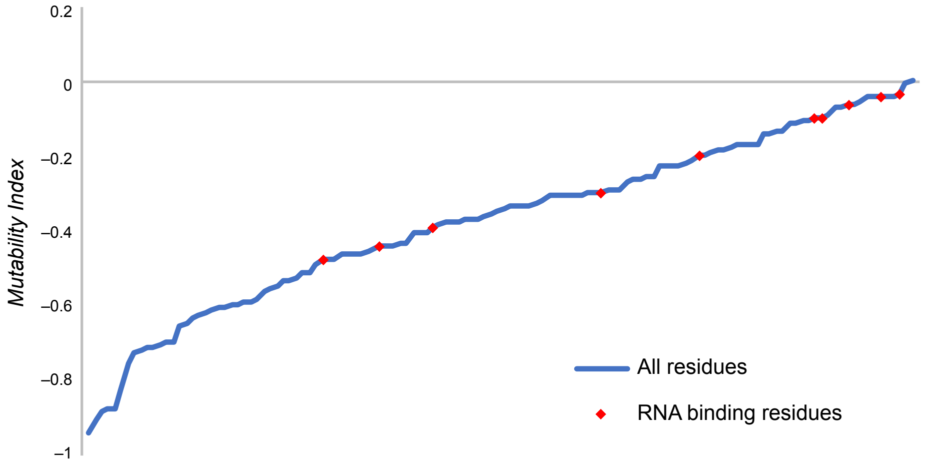

Supplementary Figure 5. Residues arranged and plotted by increasing Mutability Index. Mutability Index is the differential Shannon Entropy between the started and selected libraries and is used as a measure of permitted diversity. RNA binding residues are indicated in red.

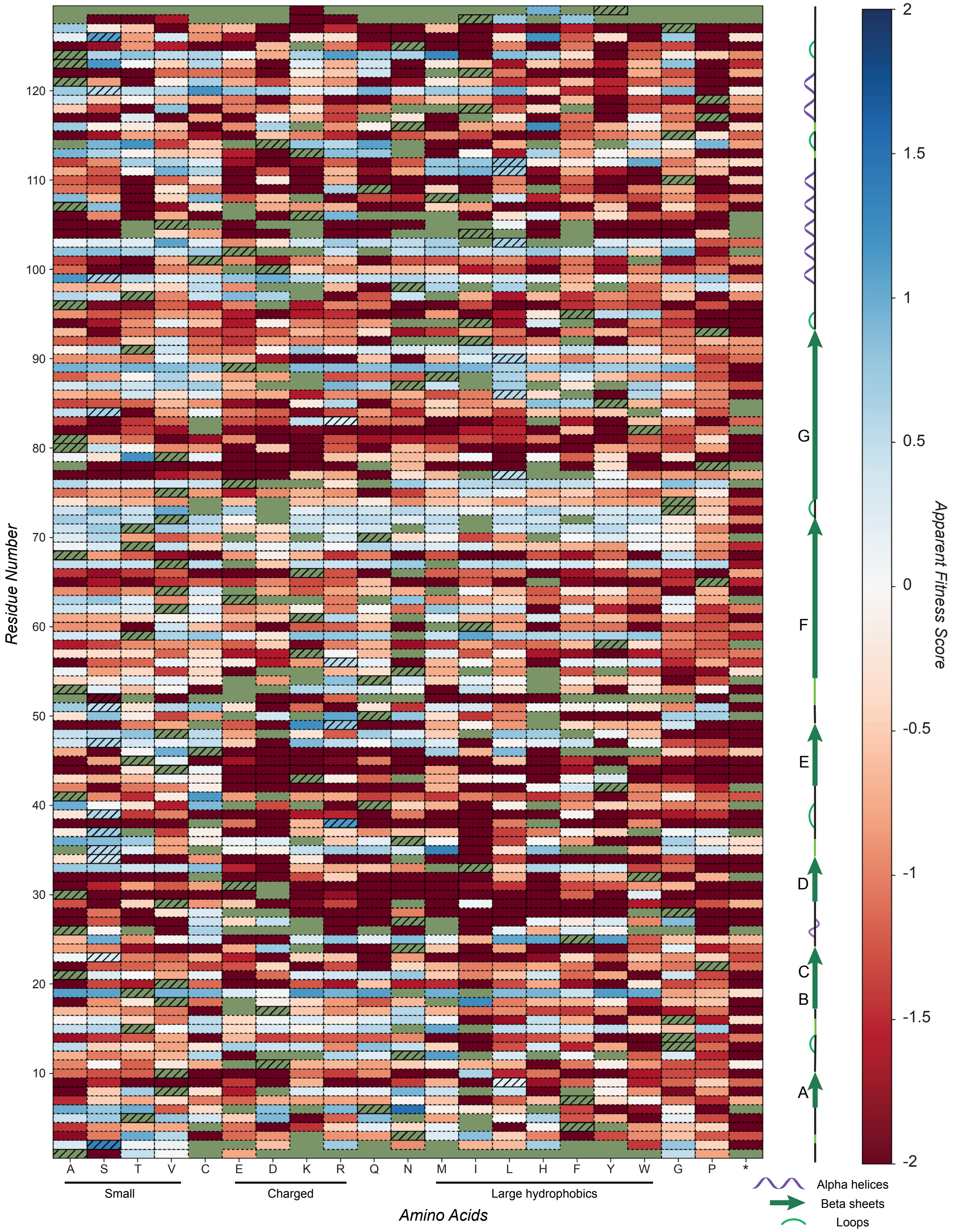

Supplementary Figure 6. Apparent Fitness Scores (AFS) where single base pair mutations are eliminated to evaluate the effects of sequencing read errors. Wild-type residues are indicated with hatches, and missing values are green. Dark red variants were sequenced before selection but absent following selection.

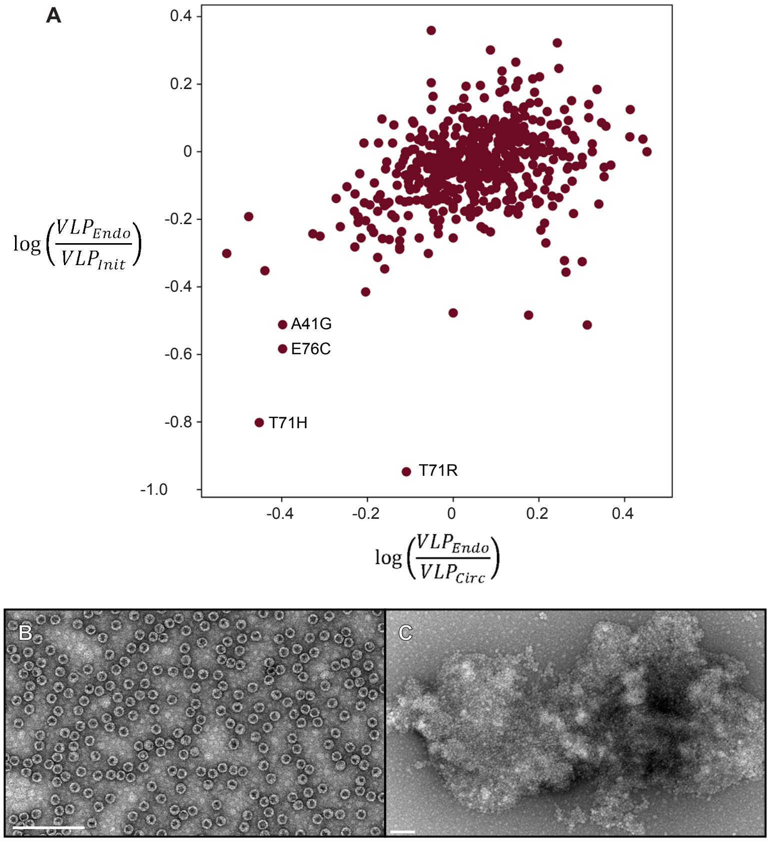

Supplementary Figure 7. Acid tolerance of MS2 CP variants. A) The population of VLPs remaining after 4 h of incubation at pH 5.0, 37 ºC (VLP_{Endo}) is compared to the population maintained at pH 7.3, 37 ºC (VLP_{Circ}) and the starting VLP library stored at pH 7.3, 4 ºC (VLP_{Init}). Variants of interest are CP[E76C] (–0.40, –0.58) and CP[T71H] (–0.45, –0.80). B) CP[WT] and C) CP[T71H/E76C] are imaged with TEM using a $UO_2(Ac)_2$ negative stain at pH 3.6. Scale bars indicate 200 nm.

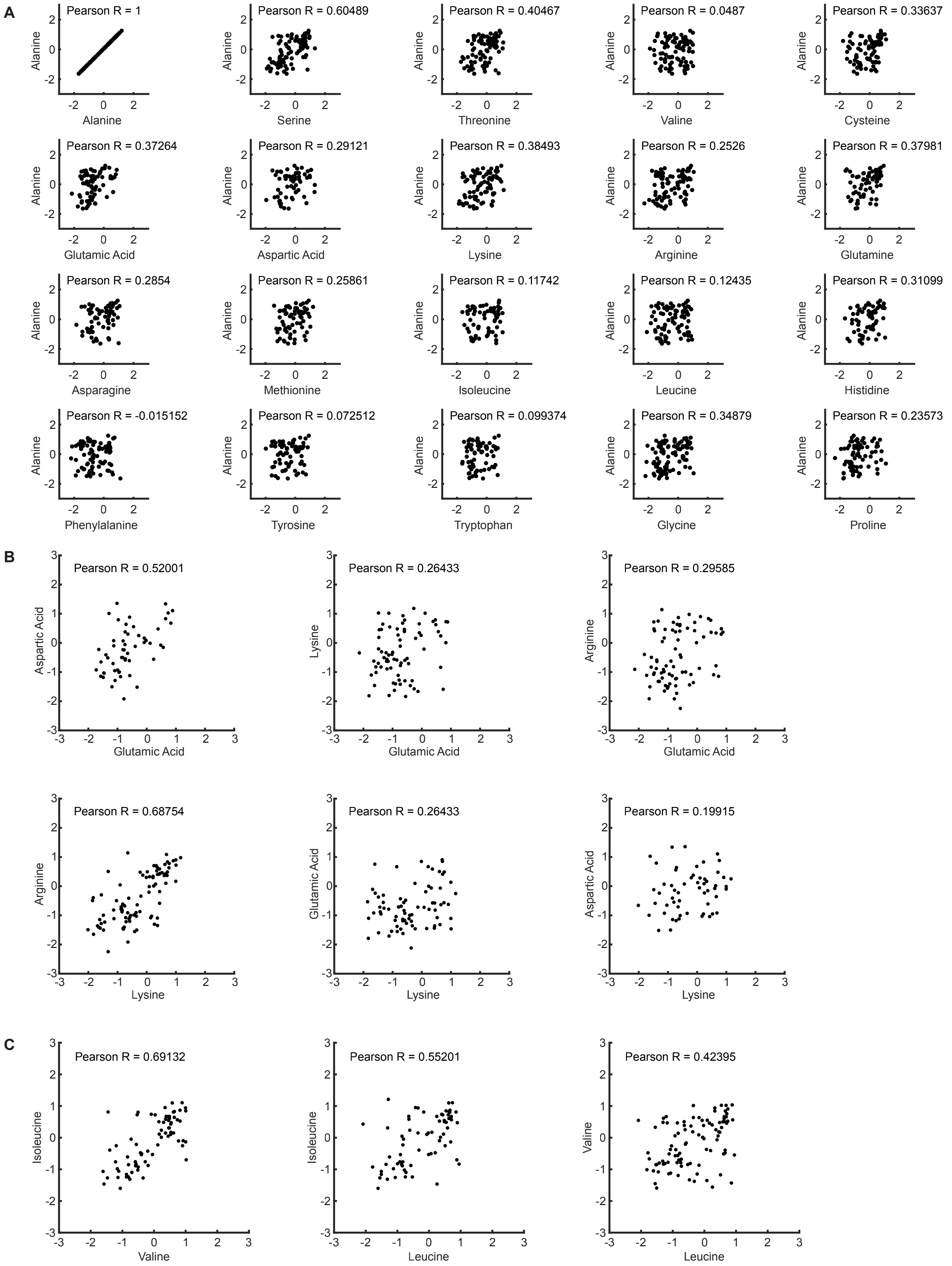

Supplementary Figure 8. AFS values correlations between A) Alanine and all other amino acids, B) Charged amino acids, and C) Selected branched, hydrophobic amino acids. Residues where the AFS value of either amino acid was –4 (sequenced before selection but not after) were excluded from this analysis.

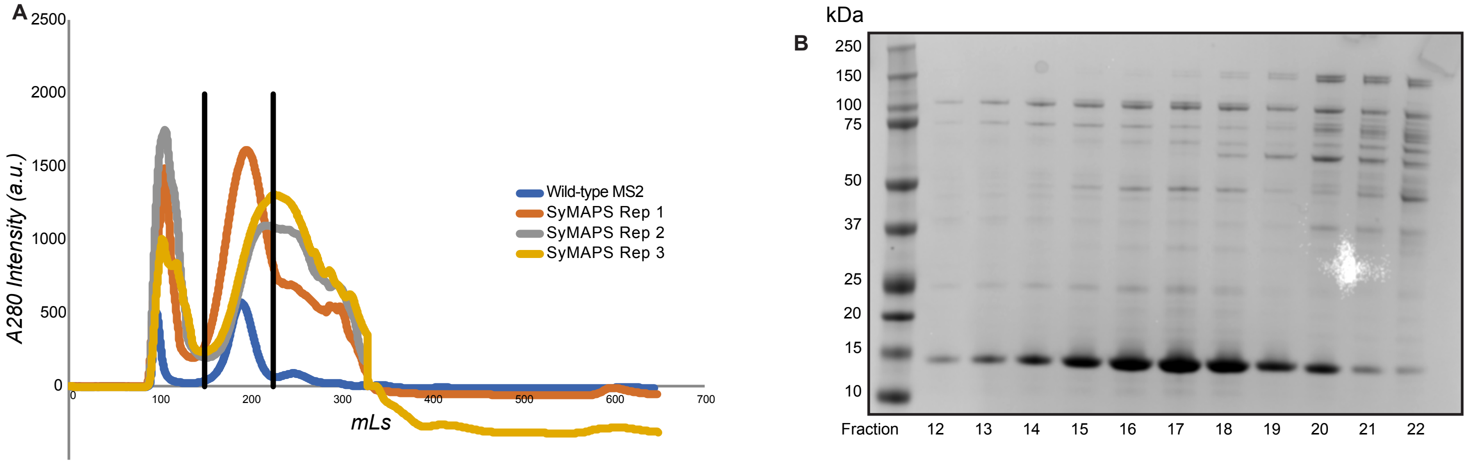

Supplementary Figure 9. SyMAPS selection using FPLC SEC. A) Traces of three SyMAPS replicates, where FPLC SEC is used to enrich for well-formed VLPs, in comparison to CP[WT]. Black lines indicate fractions that were collected for high-throughput sequencing analysis. B) SDS-PAGE gel of fractions 12 through 22 collected from CP[WT] size selection, showing enrichment for the MS2 CP band.

REFERENCES:

1. Charton, M. Protein folding and the genetic code: An alternative quantitative model. *J. Theor. Biol.* **91**, 115–123 (1981).

2. Eisenberg, D. Three-dimensional structure of membrane and surface proteins. *Ann. Rev. Biochem.* **53**, 595–623 (1984).

3. Fasman, G.D., *Handbook of Biochemistry and Molecular Biology* **1**, Cleveland: CRC Press (1976).

4. Fauchere, J.L. et al. Amino acid side chain parameters for correlation studies in biology and pharmacology. *Int. J. Peptide* Protein Res. **32**, 269–278 (1988).

5. Krigbaum, W.R. & Komoriya, A. Local interactions as a structure determinant for protein molecules: II. *Biochim. Biophys. Acta* **576**, 204–228 (1979).

6. Pontius, J. et al. Deviations from standard atomic volumes as a quality measure for protein crystal structures. *J. Mol. Biol.* **264**, 121–136 (1996).

7. Sandberg, M. et al. New chemical descriptors relevant for the design of biologically active peptides. A multivariate characterization of 87 amino acids. *J. Med. Chem.* **41**, 2481–2491. (1998).

8. Vihinen, M. et al. Accuracy of protein flexibility predictions. *Proteins* **19**, 141–149 (1994).

9. Zimmerman, J.M. et al. The characterization of amino acid sequences in proteins by statistical methods. *J. Theor. Biol.* **21**, 170–201 (1968).
