# Supplementary material for: Quantitative characterization of all single amino acid variants of a viral capsid-based drug delivery vehicle
Source: Nat Commun. 2018 Apr 11;9:1385. doi: 10.1038/s41467-018-03783-y (PMC5895741; doi:10.1038/s41467-018-03783-y)
Supplement: Supplementary file 3 — Description of Additional Supplementary Files(PDF 174 kb) [file 41467_2018_3783_MOESM3_ESM.pdf]

Description of Additional Supplementary Files

File Name: Supplementary Data 1
Description: Apparent Fitness Landscape of the MS2 CP. Residue number is indicated in column A, while amino acid identity is indicated in Row 2. “Not Observed” indicates the variant was not sequenced in the plasmid library.

File Name: Supplementary Data 2
Description: Mutability Index of the MS2 CP. Residue number is indicated in column A, and Mutability Index (Shannon Entropy plasmid – Shannon Entropy VLP) is indicated in column B.

File Name: Supplementary Data 3
Description: Mutability Index mapped onto the MS2 CP capsid. Each residue is colored according to its Mutability Index (Figure 4B).

File Name: Supplementary Data 4
Description: Primers used to generate and confirm the Apparent Fitness Landscape.
